# Exogenous 6-Benzyladenine and Kinetin Restrict Rice Seedling Root Growth, and *Ag^+^* Partially Alleviates the Inhibition in Association with Ethylene-Related Responses and Increased Lipid Peroxidation

**DOI:** 10.3390/plants15121925

**Published:** 2026-06-22

**Authors:** Xiaolong Yang, Xiaoxue Liu, Bo Li, Zeyu Li, Hanwen Yan, Yonggang He, Shuo Zhang, Zhongping Zha, Haiya Cai, Yong Fang, Ying Guo, Chunhai Jiao, Yanhao Xu

**Affiliations:** 1 Hubei Key Laboratory of Food Crop Germplasm and Genetic Improvement, Institute of Food Crops, Hubei Academy of Agricultural Sciences, Wuhan 430064, China; yxl0524@hbaas.com (X.Y.); 13227639389@163.com (X.L.); boli@hbaas.com (B.L.); lzy@hbaas.ac.cn (Z.L.); yanhanwen1994@163.com (H.Y.); whuhyg@whu.edu.cn (Y.H.); zhangshuo@hbaas.com (S.Z.); zhongpingzha@163.com (Z.Z.); caihy@hbaas.com (H.C.); guokkkyyy@163.com (Y.G.); jiaoch@hbaas.com (C.J.); 2 College of Agriculture, Yangtze University, Jingzhou 434025, China; 3 Hubei Huafengrui Agricultural Technology Co., Ltd., Wuhan 430207, China; 18007162628@163.com

**Keywords:** rice, cytokinin, 6-benzyladenine, kinetin, Ag^+^, ethylene, root architecture, lipid peroxidation, principal component analysis

## Abstract

Cytokinins are key regulators of plant root development, but their dose-dependent effects on rice seedling roots and their physiological association with ethylene-related responses remain incompletely understood. In this study, rice seedlings were exposed to two exogenous cytokinins, 6-benzyladenine (6-BA) and kinetin (KT), at different concentrations for 3 and 6 d, and Ag^+^ was used as an inhibitor of ethylene action to evaluate its alleviating effect. Both 6-BA and KT significantly inhibited primary root elongation in a concentration- and time-dependent manner. At high cytokinin concentrations, primary root length was reduced by more than 60% relative to the control, accompanied by reductions in total root length, lateral root number, absorptive area, and root vigor, as well as increased MDA and ethylene levels. Ag^+^ partially alleviated cytokinin-induced primary root inhibition, with the strongest rescue effect observed near 0.08 μM. The recovery effect was particularly evident under moderate and high cytokinin concentrations. Correlation and principal component analyses further indicated that root morphological traits were negatively associated with MDA and ethylene but positively associated with root vigor. These results suggest that exogenous cytokinins restrict rice seedling root growth through a coordinated physiological response associated with ethylene accumulation and increased membrane lipid peroxidation, while Ag^+^ partially relieves this inhibition in association with mitigation of ethylene-related restriction. Because the study was based on short-term exogenous treatments and pharmacological inhibition, the findings should be interpreted as physiological evidence for ethylene-related involvement rather than direct proof of a complete signaling mechanism.

## 1. Introduction

Root system architecture (RSA) underpins seedling establishment, early vigor, and the ability of rice plants to acquire water and nutrients from heterogeneous soil environments. In cereals, variation in primary root elongation and lateral rooting can alter both early resource capture and subsequent plant performance [1,2]. Because RSA emerges from the interaction between developmental programs, environmental inputs, and hormone signaling, it is increasingly treated as a dynamic trait rather than a fixed morphological endpoint [3,4].

Among the major hormonal regulators of RSA, cytokinins are well known for controlling meristem activity, cell differentiation, and organ patterning through a two-component signaling system [5,6,7]. In roots, cytokinin usually acts as a brake on meristem maintenance and lateral root initiation, in part by reshaping auxin distribution and auxin responsiveness [8,9,10,11,12,13]. This conceptual framework is well established in Arabidopsis, while recent studies on cytokinin regulators, sugar–cytokinin convergence, and auxin/cytokinin balance further indicate that cytokinin action is embedded in a broader hormonal network [14,15,16,17]. Auxin-related modules, including ARF and Aux/IAA components, also contribute to root morphological regulation and provide an important background for interpreting cytokinin-mediated root responses [18,19,20,21,22,23].

Early work showed that cytokinin inhibited lateral root initiation but could stimulate elongation of emerged lateral roots in rice [24]. More recent studies further indicated that supraoptimal cytokinin levels suppress primary root growth by reducing meristem size and cell length, whereas cytokinin depletion under nitrogen deficiency can promote root extension [25,26]. These reports make it necessary to compare cytokinin types and doses explicitly in rice rather than infer their effects from a single compound or a single concentration. Ethylene is another central regulator of root growth. In rice, it contributes to root plasticity under abiotic stress and root system adaptation [27,28,29,30], while in model systems, its influence on primary root growth is closely linked to receptor signaling, auxin biosynthesis, and auxin transport [31,32,33,34,35]. The ethylene precursor ACC may also have ethylene-independent functions in certain developmental contexts, adding further complexity to interpretation of ethylene-related responses [36,37]. Classical experiments showed that cytokinin-mediated inhibition of root elongation can be coupled to ethylene action [38,39], and recent work further indicates that cytokinins can regulate root growth through spatially defined ethylene production rather than through a uniform whole-root response [40]. Predictive and physiological models of cytokinin–ethylene–auxin interaction have therefore become increasingly useful for interpreting root growth phenotypes [41,42,43] This interaction is experimentally tractable because silver ions (Ag^+^) have long been used as inhibitors of ethylene action [44]. Although Ag^+^ may also affect auxin transport and therefore should be interpreted cautiously [45], Ag^+^-based assays remain useful for testing whether ethylene-related processes contribute to cytokinin responses when direct molecular validation is not yet available.

Most rice studies on hormone-regulated root growth have focused on a single regulator, one time point, or a narrow set of morphological traits. Other hormone pathways, including gibberellin- and ABA-related regulation of lateral root growth or root angle, also demonstrate that root developmental output depends on broader hormonal coordination under developmental or stress cues [46,47,48,49]. As a result, three issues remain insufficiently resolved in the cytokinin–ethylene context. First, do 6-benzyladenine (6-BA) and kinetin (KT) generate comparable dose–response patterns in rice seedlings? Second, does the magnitude of inhibition change substantially between short and longer exposure periods? Third, when cytokinin suppresses root growth, is the phenotype accompanied by coordinated shifts in root vigor, oxidative status, and ethylene level rather than by primary root length reduction alone.

To address these questions, we analyzed rice seedlings exposed to 6-BA or KT across concentration gradients for 3 and 6 d and then evaluated the extent to which Ag^+^ alleviated cytokinin-induced inhibition. We quantified primary root length, total root length, lateral root number, absorptive area, root vigor, MDA, and ethylene, and integrated these traits through correlation analysis, hierarchical clustering, and principal component analysis. Our objective was to determine not only whether exogenous cytokinin inhibits rice root growth, but also how that inhibition is organized across architectural and physiological traits. This design was intended to provide a physiological framework rather than direct molecular proof of cytokinin–ethylene signaling.

## 2. Results

### 2.1. Cytokinins Inhibit Rice Root Elongation in a Dose- and Time-Dependent Manner

**Figure 1:**
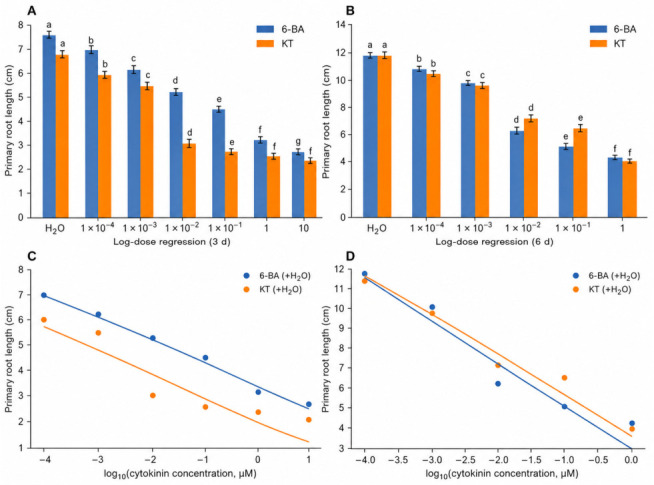
Dose-dependent inhibition of rice primary root elongation by exogenous 6-BA and KT at 3 d and 6 d. Different lowercase letters indicate significant differences among treatments within each cytokinin series at the same sampling time according to Tukey’s test. (**A**) Primary root length responses to 6-BA and KT after 3 d of treatment. (**B**) Primary root length responses to 6-BA and KT after 6 d of treatment. (**C**) Log–dose regression analysis of primary root length under 6-BA and KT treatments after 3 d of exposure. (**D**) Log–dose regression analysis of primary root length under 6-BA and KT treatments after 6 d of exposure. Data represent means of three biological replicates ± standard deviation.

As shown in Figure 1, both 6-BA and KT significantly inhibited rice primary root elongation, and the inhibitory effect became stronger with increasing concentration and prolonged treatment time. After 3 d of treatment, the mean primary root length in the H_2_O control was 7.70 cm for the 6-BA experiment and 6.79 cm for the KT experiment. Under 6-BA treatment, primary root length decreased to 6.99, 6.21, 5.30, 4.49, 3.19, and 2.78 cm at 0.0001, 0.001, 0.01, 0.1, 1, and 10 μM, respectively, corresponding to reductions of 9.3%, 19.4%, 31.2%, 41.7%, 58.6%, and 63.9% relative to the control. All concentration levels differed significantly from each other and from the control. Under KT treatment, primary root length declined to 5.99, 5.49, 3.09, 2.69, 2.49, and 2.39 cm at 0.0001, 0.001, 0.01, 0.1, 1, and 10 μM, respectively, representing reductions of 11.8%, 19.2%, 54.5%, 60.4%, 63.4%, and 64.8%. All KT treatments were significantly different from the control, whereas the difference between 1 and 10 μM KT was not significant.

The inhibitory effect became more pronounced after 6 d of exposure. In the 6-BA series, primary root length decreased from 11.88 cm in the control to 10.92, 9.94, 6.27, 5.15, and 4.30 cm at 0.0001, 0.001, 0.01, 0.1, and 1 μM, respectively, equivalent to reductions of 8.1%, 16.3%, 47.2%, 56.7%, and 63.8%. In the KT series, primary root length declined from 11.82 cm in the control to 10.64, 9.76, 7.27, 6.52, and 4.11 cm at 0.0001, 0.001, 0.01, 0.1, and 1 μM, respectively, corresponding to reductions of 10.0%, 17.4%, 38.5%, 44.9%, and 65.2%. For both cytokinins, all treatment means at 6 d were significantly different from one another and from the control. The log–dose regression further confirmed a negative relationship between cytokinin concentration and primary root length at both sampling times. These results indicate that cytokinin-induced primary root growth inhibition was both concentration-dependent and time-dependent, with KT showing a slightly stronger inhibitory tendency than 6-BA at the highest concentration range.

### 2.2. Cytokinin Treatment Reshapes Rice Root Architecture

**Figure 2:**
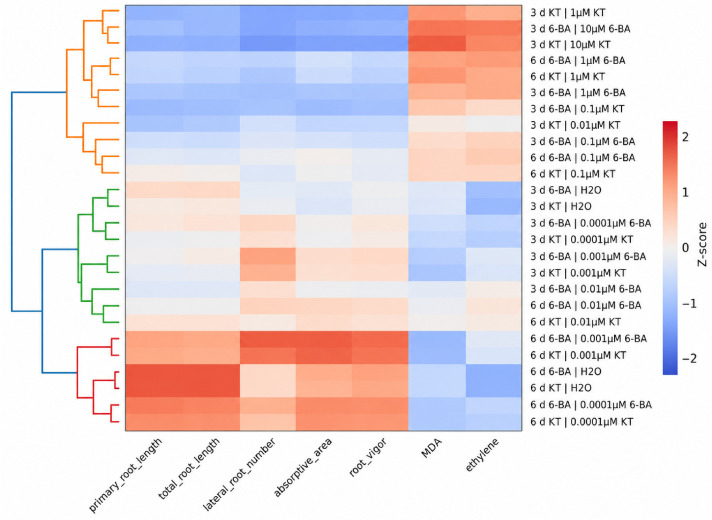
Hierarchical clustering heatmap of standardized morphological and physiological traits under cytokinin-only treatments. The heatmap shows the standardized means of root morphological and physiological variables measured under 6-BA and KT treatments at 3 d and 6 d. Red indicates relatively higher trait values, whereas blue indicates relatively lower trait values after row-wise standardization.

To further integrate the treatment responses, the standardized means of root morphological and physiological traits under cytokinin-only treatments were subjected to hierarchical clustering (Figure 2). The heatmap resolved the treatments into two major response domains. One domain comprised the control and low-cytokinin treatments, which were characterized by relatively higher lateral root number, absorptive area, and root vigor, together with comparatively lower MDA and ethylene levels. The other domain was dominated by high-cytokinin treatments and was associated with reduced primary root length, total root length, lateral root number, and absorptive area, accompanied by elevated MDA and ethylene.

This clustering pattern indicates that the effects of cytokinin on rice roots were not simply linear reductions in all traits. At low concentrations, especially around 0.0001–0.001 μM, cytokinin treatment caused only limited restriction of the main root axis, while some architectural traits, including lateral root number and absorptive area, were maintained or even enhanced relative to the control. In contrast, with increasing cytokinin concentration, the response shifted toward a collapsed inhibitory state in which both morphological development and physiological status deteriorated simultaneously. This transition was evident for both 6-BA and KT, and became more pronounced at 6 d than at 3 d, indicating a stronger coupling between architectural restriction and physiological stress under prolonged cytokinin exposure.

### 2.3. Ag^+^ Alleviates Cytokinin-Induced Root Growth Inhibition

**Figure 3:**
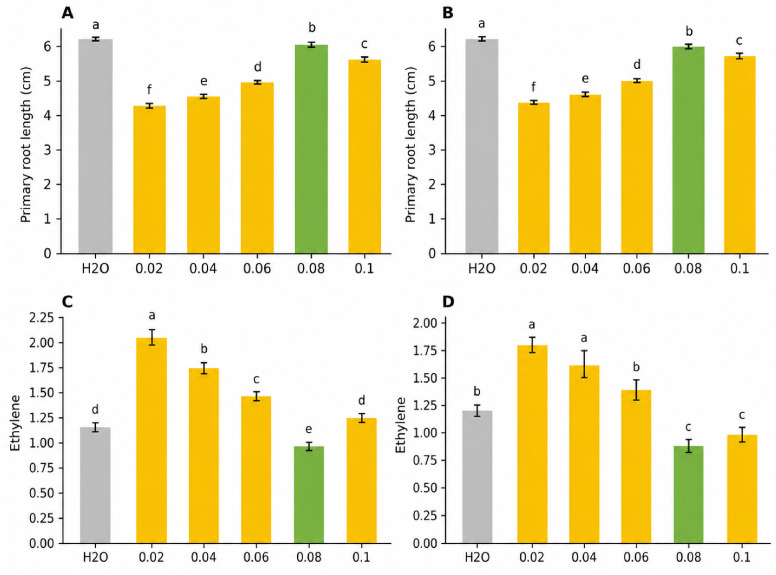
Effects of the *Ag^+^* gradient under 0.01 μM cytokinin at 3 d. Different lowercase letters indicate significant differences among treatments within each experiment according to Tukey’s test. (**A**) primary root length in the 6-BA background. (**B**) primary root length in the KT background. (**C**) Ethylene in the 6-BA background. (**D**) Ethylene in the KT background. Ag^+^ partially alleviated cytokinin-induced root inhibition in a concentration-dependent manner, with the strongest rescue observed near 0.08 μM in both cytokinin backgrounds. Data represent means of three biological replicates ± standard deviation.

As shown in Figure 3, the alleviating effect of Ag^+^ on cytokinin-inhibited primary root growth at 3 d was strongly concentration-dependent in both the 6-BA and KT backgrounds, and the response generally followed a bell-shaped pattern with the greatest primary root length observed at 0.08 μM Ag^+^. In the 6-BA background, the mean primary root length in the H_2_O control was 6.28 cm. Under combined treatment with 0.01 μM 6-BA and Ag^+^, primary root length was 4.33, 4.58, 5.03, 6.10, and 5.70 cm at 0.02, 0.04, 0.06, 0.08, and 0.1 μM Ag^+^, respectively, corresponding to reductions of 31.1%, 27.1%, 19.9%, 2.8%, and 9.2% relative to the control. All treatment means differed significantly from one another and from the H_2_O control. Compared with the 0.01 μM 6-BA treatment alone in Figure 1 (5.30 cm), only 0.08 and 0.1 μM Ag^+^ increased primary root length, by 15.1% and 7.5%, respectively, whereas 0.02–0.06 μM Ag^+^ did not restore primary root growth to the cytokinin-only level.

A similar concentration-dependent pattern was observed in the KT background, but the rescuing effect of Ag^+^ was more pronounced. The H_2_O control had a mean primary root length of 6.19 cm. Under combined treatment with 0.01 μM KT and Ag^+^, primary root length reached 4.38, 4.59, 4.99, 5.99, and 5.79 cm at 0.02, 0.04, 0.06, 0.08, and 0.1 μM Ag^+^, respectively, corresponding to reductions of 29.2%, 25.8%, 19.4%, 3.2%, and 6.5% relative to the control. All KT + Ag^+^ treatments were significantly different from one another and from the H_2_O control. Compared with the 0.01 μM KT treatment alone in Figure 1 (3.09 cm), the addition of Ag^+^ increased primary root length by 41.7%, 48.5%, 61.5%, 93.8%, and 87.4% at 0.02, 0.04, 0.06, 0.08, and 0.1 μM, respectively. In both cytokinin backgrounds, 0.08 μM Ag^+^ produced the strongest alleviating effect, whereas a further increase to 0.1 μM led to a slight but significant decline in primary root length. These results indicate that Ag^+^ partially alleviated cytokinin-induced primary root growth inhibition at 3 d, with a clearly defined concentration optimum around 0.08 μM.

### 2.4. *Ag^+^* Improves Root Physiological Status Under Cytokinin Stress

**Figure 4:**
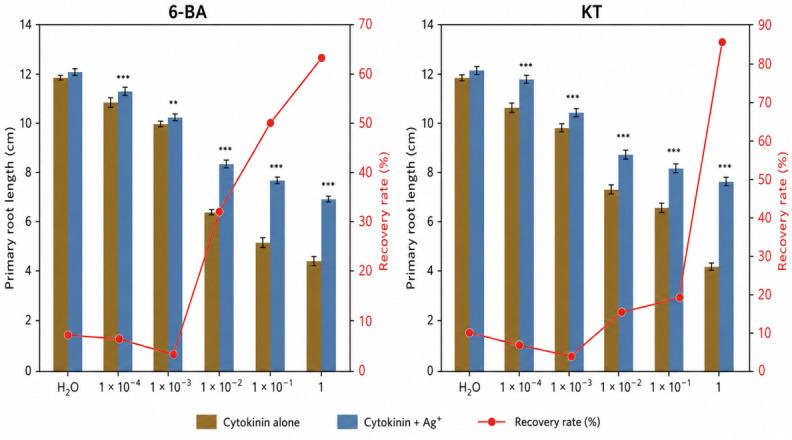
Primary root length rescue by 0.08 μM Ag^+^ at 6 d. Bars indicate cytokinin-only and cytokinin + Ag^+^ treatments, and the red line denotes recovery percentage. The addition of 0.08 μM Ag^+^ partially restored cytokinin-inhibited primary root elongation across all tested cytokinin concentrations, with stronger relative recovery at moderate and high cytokinin doses. Asterisks indicate significant differences between paired cytokinin-only and cytokinin + Ag^+^ values within the same concentration (** *p* < 0.01, *** *p* < 0.001).

As shown in Figure 4, the addition of 0.08 μM Ag^+^ partially restored cytokinin-inhibited primary root growth after 6 d of exposure, although the extent of recovery depended on the cytokinin concentration. In the 6-BA series, the mean primary root length in the H_2_O control was 12.00 cm. Under combined treatment with 6-BA and 0.08 μM Ag^+^, primary root length was 11.26, 10.15, 8.29, 7.66, and 6.95 cm at 0.0001, 0.001, 0.01, 0.1, and 1 μM 6-BA, respectively, corresponding to reductions of 6.2%, 15.4%, 30.9%, 36.2%, and 42.1% relative to the control. Compared with the corresponding 6-BA-only treatments shown in Figure 1, primary root length increased from 10.92 to 11.26 cm, from 9.94 to 10.15 cm, from 6.27 to 8.29 cm, from 5.15 to 7.66 cm, and from 4.30 to 6.95 cm, representing recovery rates of 3.1%, 2.1%, 32.2%, 48.8%, and 61.7%, respectively. All combined-treatment means were significantly different from one another and from the H_2_O control, and the addition of 0.08 μM Ag^+^ significantly increased primary root length relative to the corresponding 6-BA-only treatments.

A similar but slightly stronger rescue pattern was observed in the KT series. The mean primary root length in the H_2_O control was 12.12 cm. Under combined treatment with KT and 0.08 μM Ag^+^, primary root length reached 11.74, 10.37, 8.65, 8.04, and 7.65 cm at 0.0001, 0.001, 0.01, 0.1, and 1 μM KT, respectively, corresponding to reductions of 3.1%, 14.4%, 28.6%, 33.7%, and 36.9% relative to the control. Compared with the corresponding KT-only treatments in Figure 1, primary root length increased from 10.64 to 11.74 cm, from 9.76 to 10.37 cm, from 7.27 to 8.65 cm, from 6.52 to 8.04 cm, and from 4.11 to 7.65 cm, corresponding to increases of 10.3%, 6.3%, 19.0%, 23.3%, and 86.2%, respectively. For the KT series, all combined-treatment means were also significantly different from one another and from the control. Notably, the relative recovery became more pronounced as cytokinin concentration increased, especially at 1 μM, where Ag^+^ markedly alleviated the inhibitory effect in both cytokinin backgrounds. These results indicate that 0.08 μM Ag^+^ partially counteracted cytokinin-induced primary root growth inhibition at 6 d, with the rescue effect being particularly evident under moderate and high cytokinin concentrations.

### 2.5. Multivariate Analysis Distinguishes Cytokinin Stress from *Ag^+^* Rescue

Across integrated 6 d treatment means, primary root length was strongly correlated with total root length (r = 0.995), root vigor (r = 0.856), and absorptive area (r = 0.817). By contrast, primary root length was negatively correlated with MDA (r = −0.869) and ethylene (r = −0.885). MDA and ethylene were positively associated with each other and negatively associated with growth-promoting traits, indicating that architectural impairment, increased membrane lipid peroxidation, and ethylene accumulation formed a coherent response syndrome rather than independent treatment effects. The strong association between primary root length and total root length is expected because total root length includes the primary root as one component; therefore, these two traits are related but not mathematically identical.

**Figure 5:**
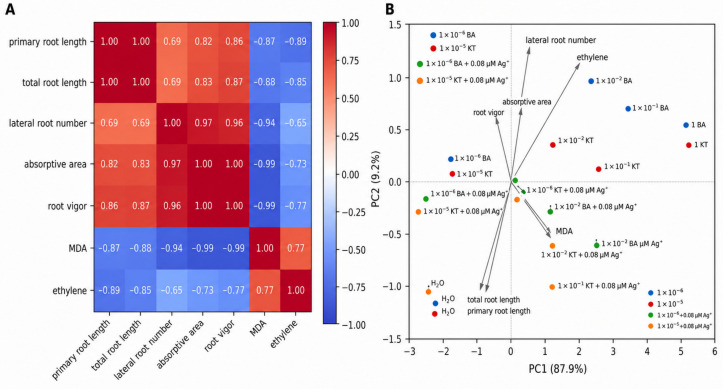
Integrative multivariate analyses of 6 d treatments. (**A**) Correlation heatmap of treatment means. (**B**) PCA biplot showing separation of cytokinin-only and Ag^+^ rescued treatments and the direction of trait loadings.

PCA reinforced this interpretation. PC1 explained 88.0% of the total variance and separated high-dose cytokinin-only treatments from low-dose and Ag^+^-rescued treatments. Variables with positive loadings on PC1 included primary root length, total root length, absorptive area, and root vigor, whereas MDA and ethylene loaded in the opposite direction (Figure 5). Together with the clustering result, the PCA shows that Ag^+^ shifted treatment profiles away from the high-ethylene/high-MDA inhibitory state toward a more growth-competent physiological state. However, this shift should be interpreted as physiological association rather than direct proof of a specific signaling pathway.

Two-way ANOVA further confirmed that cytokinin concentration was the dominant driver of variation across the single-cytokinin datasets (Table 1). At 3 d, concentration effects were highly significant for primary root length (F = 13,293.67, *p* < 0.001), total root length (F = 3226.84, *p* < 0.001), root vigor (F = 2163.60, *p* < 0.001), MDA (F = 672.86, *p* < 0.001), and ethylene (F = 691.43, *p* < 0.001). The corresponding main effects of the hormone were also significant, but generally smaller in magnitude. At 6 d, the concentration effect intensified further, especially for primary root length (F = 59,526.26, *p* < 0.001), total root length (F = 7658.11, *p* < 0.001), and root vigor (F = 3883.03, *p* < 0.001). Significant hormone × concentration interactions were detected for primary root length and total root length at both sampling times, whereas the interaction terms for lateral root number, root vigor, MDA, and ethylene were not significant at 6 d, indicating that the divergence between 6-BA and KT was strongest for elongation-related traits rather than for the broader stress-response variables.

The regression summaries were consistent with this interpretation (Table 2). Primary root length showed a strong negative log–dose relationship for both cytokinins at 3 d and 6 d, with slopes of −0.882 and −0.783 at 3 d for 6-BA and KT, respectively, and steeper slopes of −1.803 and −1.630 at 6 d. The model fits were high (R^2^ = 0.823–0.989; *p* < 0.001), confirming a robust concentration-dependent decline in primary root growth. By contrast, ethylene showed positive log–dose relationships under both cytokinins, with slopes of 0.509–0.520 and R^2^ values of 0.986–0.990 (*p* < 0.001). Together, these analyses indicate that increasing cytokinin concentration progressively suppressed primary root elongation while simultaneously enhancing ethylene accumulation, and that both responses became more pronounced after prolonged exposure.

Consistent with the paired comparisons shown in Figure 4, the recovery percentages indicated that the rescuing effect of 0.08 μM Ag^+^ was minimal at 0.0001–0.001 μM cytokinin (1.5–11.0%), but increased sharply at moderate and high cytokinin concentrations. In the 6-BA series, recovery reached 30.8%, 47.1%, and 44.8% at 0.01, 0.1, and 1 μM, respectively, whereas in the KT series the corresponding values were 24.7%, 58.0%, and 92.3%. These values further support that Ag^+^ exerted its strongest relative rescue when cytokinin-induced inhibition was severe, particularly in the KT background.

### 2.6. Working Model of Cytokinin-Induced Root Inhibition and Ag^+^ Alleviation

**Figure 6:**
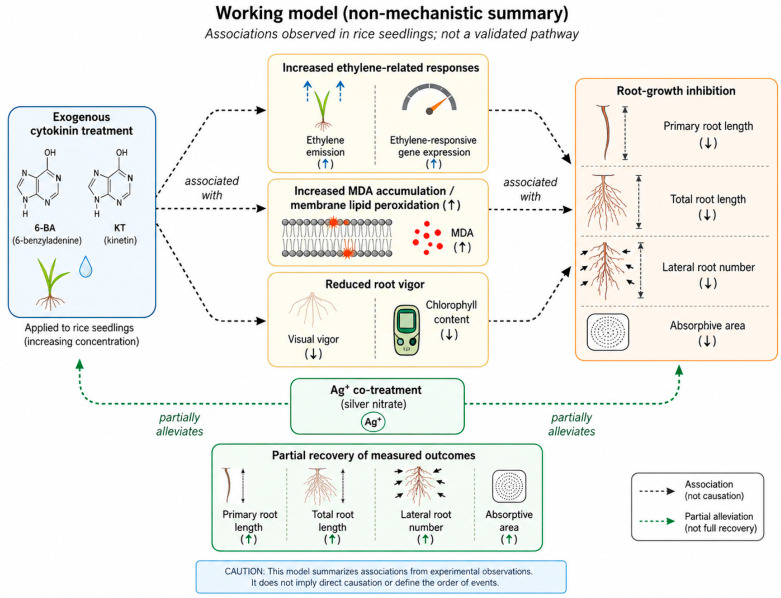
Working model summarizing cytokinin-induced root inhibition and Ag^+^-associated alleviation in rice seedlings. Exogenous cytokinin treatment (6-BA or KT) induced a dose- and time-dependent inhibitory phenotype in rice roots, characterized by reduced primary root length, total root length, lateral root number, and absorptive area. These morphological changes were accompanied by increased MDA accumulation, which indicates enhanced membrane lipid peroxidation, reduced root vigor, and enhanced ethylene-related response. Ag^+^ treatment, with the strongest alleviating effect observed near 0.08 μM, partially reversed the cytokinin-induced inhibitory phenotype, resulting in improved root growth and architecture, decreased MDA levels, and increased root vigor. This schematic model summarizes the coordinated morphological and physiological responses observed in the present study and provides a working model for interpreting the Ag^+^-associated alleviation of cytokinin-induced root growth inhibition. In the schematic diagram, black dashed arrows indicate associations based on the observed morphological and physiological responses, green dashed arrows indicate the partial alleviation associated with Ag^+^ co-treatment, and upward or downward arrows indicate an increase or decrease in the corresponding trait or physiological index.

Figure 6 provides a working model summarizing the morphological and physiological responses of rice seedlings to exogenous cytokinin treatment and Ag^+^-rescue. In the absence of cytokinin, rice seedlings exhibited normal root development, including greater primary root length, total root length, lateral root number, and absorptive area. By contrast, exogenous cytokinin treatment (6-BA or KT) induced a dose- and time-dependent inhibitory phenotype, which was characterized by shorter primary roots, lower total root length, lower lateral root number, and a smaller absorptive area. These morphological changes were accompanied by increased MDA accumulation, which indicates enhanced membrane lipid peroxidation, and decreased root vigor. The integrative analysis further suggested that cytokinin treatment was associated with an enhanced ethylene-related response. Under these conditions, Ag^+^ treatment, with the strongest alleviating effect observed near 0.08 μM, partially reversed the cytokinin-induced inhibition. Seedlings exposed to cytokinin plus Ag^+^ showed longer primary roots, improved total root length, higher lateral root number, and a larger absorptive area than those under cytokinin treatment alone. In parallel, MDA content decreased and root vigor increased after Ag^+^ application. Therefore, Figure 6 should be regarded as a working model based on morphological, physiological, and pharmacological evidence, rather than as a validated molecular pathway.

## 3. Discussion

Root system establishment during the early seedling stage is a critical determinant of subsequent nutrient acquisition, water uptake, and stress adaptation in rice, because the primary and newly formed lateral roots largely define the initial absorptive architecture of the plant. Root development is highly plastic and strongly regulated by hormonal crosstalk, among which cytokinin, auxin, and ethylene form an important regulatory network controlling root meristem activity, lateral roots formation, and stress-responsive architectural adjustment [4,5,8,27,32,33]. Although cytokinin has long been recognized as an important regulator of meristem maintenance and cell differentiation [5,6,7,9,10], the quantitative inhibitory threshold of exogenous cytokinins on rice seedling roots and its physiological association with ethylene-related responses remain insufficiently resolved. In addition, direct evidence describing whether the cytokinin-induced inhibitory phenotype can be mitigated by Ag^+^ under a concentration-dependent framework is still scarce. Therefore, the present study aimed to establish an integrated morphological and physiological dataset to characterize cytokinin-mediated root inhibition and Ag^+^-associated alleviation during early rice root development.

The present results clearly showed that both 6-BA and KT exerted marked inhibitory effects on rice primary root elongation, and the degree of inhibition increased progressively with cytokinin concentration and exposure duration (Figure 1). In particular, primary root length at the high-concentration range declined by more than 60% relative to the control, indicating that rice roots were highly sensitive to excessive cytokinin input. This result is consistent with previous reports that elevated cytokinin levels restrict root apical meristem activity, reduce cell proliferation, and interfere with longitudinal root expansion [9,10,11,12,13,14,15,24,25,26]. Cytokinin–ethylene crosstalk may further contribute to this inhibitory output by promoting ethylene biosynthesis or signaling [38,39,40,41,42]. This interpretation is supported by our clustering analysis, in which the high-cytokinin treatments were consistently grouped with elevated ethylene and MDA levels but reduced root vigor and absorptive traits (Figure 2; Table 1). Thus, cytokinin-induced inhibition in the present work should not be interpreted as a simple decrease in primary root length alone; rather, it represented a coordinated high-ethylene/high-MDA restrictive state accompanied by deterioration of root physiological activity. Because MDA is a downstream marker of lipid peroxidation, the present data support increased membrane lipid peroxidation rather than directly proving ROS generation or a complete oxidative-stress pathway. Moreover, the stronger separation of the high-dose response domain at 6 d than at 3 d (Figure 2) indicates that prolonged cytokinin exposure gradually shifted the seedlings from mild architectural adjustment toward a more severe stress-associated inhibition syndrome. No direct ROS quantification, such as ROS-specific staining or antioxidant enzyme profiling, was performed; therefore, MDA is discussed here as a lipid-peroxidation marker rather than as direct evidence of ROS generation.

Another notable finding of this study is that Ag^+^ substantially alleviated cytokinin-induced root restriction, but the rescue effect was highly dependent on Ag^+^ concentration and cytokinin background. As shown in Figure 3, Ag^+^ produced a bell-shaped recovery pattern at 3 d, with the strongest restoration occurring near 0.08 μM, indicating that the antagonistic effect of Ag^+^ operated within an optimal concentration window rather than through a linear stimulatory response. When 0.08 μM Ag^+^ was subsequently applied across increasing cytokinin concentrations, the relative recovery became progressively greater under moderate and severe cytokinin inhibition, particularly in the 1 μM treatments (Figure 4). Since *Ag^+^* is widely used as an inhibitor of ethylene perception [44], these observations imply that a substantial portion of the cytokinin inhibitory phenotype observed here was associated with an ethylene-related restriction pathway. This inference should be interpreted cautiously because Ag^+^ is a silver ion and may cause heavy-metal-associated effects or influence auxin efflux independently of ethylene response [45]. The partial nature of the rescue also suggests that ethylene-independent pathways, such as direct cytokinin effects, auxin redistribution, or ABA-related responses, may contribute to the remaining inhibition. Therefore, the present Ag^+^ response pattern provides pharmacological and physiological evidence for ethylene-related involvement, but it should not be taken as direct mechanistic proof of ethylene-mediated signaling [33,35,36,37,38,39].

The coordinated multivariate relationships among morphological and physiological traits further strengthen this interpretation. Correlation analysis and principal component separation revealed that primary root length, total root length, lateral root number, and absorptive area were consistently negatively associated with MDA and ethylene, whereas root vigor showed a strong positive association with the maintenance of root architectural integrity (Figure 5). This indicates that cytokinin treatment simultaneously influenced root structural development, membrane lipid peroxidation status, and metabolic activity, and these variables should therefore be considered as components of one integrated response network rather than isolated parameters. Based on this convergent evidence, the working model summarized in Figure 6 proposes that exogenous cytokinin likely triggered an ethylene-associated inhibitory syndrome characterized by suppressed primary root elongation, reduced absorptive architecture, enhanced lipid peroxidation, and decreased root vigor, whereas Ag^+^ partially disrupted this coordinated restriction and restored root developmental performance. Although the present study does not directly verify the molecular signaling nodes linking cytokinin and ethylene, current evidence from hormone crosstalk studies indicates that cytokinin can enhance ethylene biosynthetic flux and thereby reshape root growth output through downstream hormonal coordination [38,39,40,41,42,43]. Therefore, our results provide a quantitative physiological framework for interpreting cytokinin–ethylene association in early rice root development and help explain why Ag^+^ exerts a concentration-dependent alleviating effect on cytokinin-induced root growth restriction.

Several limitations of the present experimental approach should be acknowledged. First, the study used exogenous 6-BA and KT to establish a controlled dose–response framework. Although this design is useful for comparing cytokinin types and concentrations under defined conditions, high-dose exogenous application may induce pharmacological responses that do not fully represent endogenous cytokinin regulation under field conditions. In addition, 6-BA is a synthetic cytokinin, whereas rice plants mainly rely on endogenous cytokinin forms such as trans-zeatin and N^6^-(Δ^2^-isopentenyl) adenine. Therefore, the present results should be interpreted as responses to exogenous cytokinin analogs rather than as direct evidence of native cytokinin homeostasis. Second, the observations were limited to early seedling responses after 3 and 6 d of treatment in a solution–culture system. Root architecture, hormone sensitivity, ethylene diffusion, and nutrient uptake capacity may differ under soil conditions and at later developmental stages, such as tillering and reproduction. Moreover, although reduced absorptive area and root vigor suggest a possible decline in nutrient acquisition potential, nitrogen and phosphorus uptake were not directly measured. Finally, the present study relied mainly on morphological traits, root vigor, MDA content, ethylene measurements, and Ag^+^-based pharmacological alleviation. It did not directly quantify ROS accumulation, antioxidant enzyme activity, endogenous hormone levels, or the expression of cytokinin- and ethylene-related genes, nor did it use genetic materials to verify the proposed regulatory relationships. These limitations mean that the current findings should be regarded as physiological and pharmacological evidence for cytokinin-associated root growth inhibition and Ag^+^-associated alleviation, rather than as direct molecular proof of a complete cytokinin–ethylene signaling pathway.

## 4. Materials and Methods

### 4.1. Plant Material, Seed Germination, and Chemical Reagents

The pot experiment was conducted in 2025 at the pot-culture facility of the Nanhu Experimental Base, Hubei Academy of Agricultural Sciences, Wuhan, Hubei Province, China. The Huanghuazhan rice cultivate was used in all experiments. The cytokinin treatments comprised 6-benzyladenine (6-BA) and kinetin (KT), and AgNO_3_ was used as the source of Ag^+^. Sodium hypochlorite solution was used for seed surface sterilization. Seeds were surface-sterilized in 5% (*v*/*v*) sodium hypochlorite for 15 min, rinsed thoroughly with distilled water, soaked for 24 h, and pre-germinated for an additional 24 h. Uniformly germinated seeds with comparable radicle emergence were selected for subsequent treatments.

### 4.2. Treatment Design and Seedling Culture

For the single-cytokinin assays, germinated seeds were cultured in 400 mL of treatment solution containing either distilled water (H_2_O control) or a concentration series of 6-BA or KT. The 3 d assays included 0.0001–10 μM cytokinin, whereas the 6 d assays included 0.0001–1 μM cytokinin. For the Ag^+^ gradient assays, seedlings were exposed to 0.01 μM KT or 0.01 μM 6-BA in combination with 0.02, 0.04, 0.06, 0.08, or 0.10 μM Ag^+^. For the 0.08 μM Ag^+^ rescue assays, cytokinin concentration series were combined with 0.08 μM Ag^+^ and evaluated after 3 or 6 d. Each treatment included three biological replicates, with 10–12 seedlings per replicate. Seedlings were maintained at 28 ± 2 °C under a 16 h light/8 h dark photoperiod, with a relative humidity of 75% and a light intensity of 400 μmol m^−2^ s^−1^. Treatment solutions were renewed during prolonged incubation to minimize concentration drift and secondary nutrient depletion. Because these experiments were conducted in a solution–culture system, hormone availability, root-zone aeration, and ethylene diffusion may differ from those in soil-grown plants; this limitation was considered when interpreting the physiological relevance of the results.

### 4.3. Root Imaging and Quantification of Root Morphological Traits

At each sampling time, seedlings from each biological replicate were gently removed from the treatment solution, and the roots were rinsed with distilled water to eliminate residual solution adhering to the root surface. For morphological analysis, intact root systems were carefully spread in a shallow transparent tray containing a thin layer of water to minimize overlap among root segments. Whole-root images were then acquired under uniform illumination and analyzed using an image-based root phenotyping workflow adapted from established 2D root imaging studies [50,51]. Primary root length was defined as the length of the main root derived from the radicle at the seedling stage. Total root length was calculated as the summed length of all visible root segments, including the primary root and lateral roots; lateral root number was counted manually from the digitized images, and absorptive area was extracted as projected root area from the same images. For each biological replicate, trait values were averaged across all seedlings included in that replicate before statistical analysis.

### 4.4. Measurement of Root Vigor, MDA, and Ethylene

Root vigor was determined by the triphenyl tetrazolium chloride (TTC) reduction assay following the principle described by Steponkus and Lanphear [52] with minor adaptation for rice seedling roots. Briefly, fresh root samples were blotted dry, weighed, and incubated in a reaction mixture containing 0.4% (*w*/*v*) TTC and phosphate buffer (pH 7.0) in the dark at 37 °C. The reaction was terminated with 1 mol L^−1^ H_2_SO_4_, and the reduced triphenyl formazan was extracted with ethyl acetate. Absorbance was then determined spectrophotometrically at 485 nm, and root vigor was expressed as μg TTC reduced g^−1^ fresh weight h^−1^.

Malondialdehyde (MDA) content was used as an indicator of membrane lipid peroxidation and was determined using the thiobarbituric acid (TBA) reaction according to the classical method of Heath and Packer [53]. Fresh root tissue was homogenized in ice-cold 0.1% (*w*/*v*) trichloroacetic acid, and the homogenate was centrifuged to obtain the supernatant. An aliquot of the supernatant was mixed with 0.5% (*w*/*v*) TBA prepared in 20% (*w*/*v*) trichloroacetic acid, heated in a boiling water bath, rapidly cooled, and centrifuged again. The absorbance of the supernatant was recorded at 532 nm and corrected for nonspecific turbidity at 600 nm. MDA concentration was expressed as nmol g^−1^ fresh weight.

Ethylene was quantified as headspace ethylene concentration following published gas chromatography-based seedling protocols [54,55]. For each replicate, uniformly treated seedlings were transferred into sealed vials containing the corresponding treatment solution and incubated for a fixed accumulation period under the same growth conditions used for the bioassay. A defined volume of headspace gas was then withdrawn with a gas-tight syringe and analyzed by gas chromatography. Ethylene concentration was expressed as μL L^−1^ headspace. In the present study, these ethylene values were used as an index of ethylene-associated response intensity under cytokinin and Ag^+^ treatments.

### 4.5. Data Processing and Recovery Calculation

For the 0.08 μM Ag^+^ rescue datasets, the spreadsheet contained primary root length values for cytokinin-treated seedlings and the corresponding primary root length values after Ag^+^ rescue under the same cytokinin concentration. Recovery percentage was calculated as ((primary root length after Ag^+^ rescue − primary root length before Ag^+^ rescue)/primary root length before Ag^+^ rescue) × 100. For multivariate analyses, treatment means were standardized to Z scores prior to hierarchical clustering, correlation analysis, and principal component analysis (PCA).

### 4.6. Statistical Analysis

All data are presented as means ± standard deviation (SD) of three biological replicates. One-way ANOVA followed by Tukey’s honestly significant difference (HSD) test was used to compare treatments within each independent experiment. Two-way ANOVA was applied to the single-cytokinin datasets to test the effects of hormone identity, concentration, and their interaction. For the rescue datasets, paired t-tests were used to compare primary root length before and after Ag^+^ rescue at the same cytokinin concentration. Log–dose regression was fitted by linear regression of response variables against log10-transformed cytokinin concentration. Hierarchical clustering and correlation analyses were performed using standardized treatment means, and PCA was conducted on the same standardized dataset. Different lowercase letters in the figures indicate significant differences among treatments within the same experiment at *p* < 0.05. Individual seedlings within each biological replicate were first averaged, and statistical tests were conducted using biological replicate means; individual seedlings were not treated as independent statistical replicates.

## 5. Conclusions

This study shows that exogenous 6-BA and KT restrict rice seedling root growth in a dose- and time-dependent manner, with concentration exerting the strongest overall effect in the single-cytokinin datasets. The response was not limited to primary root shortening: low doses could transiently reshape root system architecture, whereas higher doses were consistently associated with reduced root vigor and elevated MDA and ethylene. Ag^+^ partially alleviated cytokinin-induced inhibition, and the rescue was strongest at 0.08 μM in the present experiments. Taken together, the data indicate that rice root restriction by exogenous cytokinins is best understood as a coordinated architectural and physiological response associated with ethylene-related responses and increased membrane lipid peroxidation rather than a single-trait effect. Because the present evidence for ethylene involvement is based on physiological and pharmacological observations, the relationship should be interpreted cautiously. Future studies combining endogenous hormone profiling, nutrient-uptake measurements, OsACS/OsACO and OsHK/OsRR expression analyses, and genetic materials will be needed to test the proposed regulatory links under soil and full-cycle growth conditions.

## Figures and Tables

**Table 1:** Two-way ANOVA summary for the single-cytokinin datasets.

Time	Trait	Source	F	*p*
3 d	Primary root length	Hormone	8380.81	<0.001
		Concentration	13,293.67	<0.001
		Hormone × concentration	417.79	<0.001
	Total root length	Hormone	1372.00	<0.001
		Concentration	3226.84	<0.001
		Hormone × concentration	84.88	<0.001
	Lateral root number	Hormone	54.32	<0.001
		Concentration	237.56	<0.001
		Hormone × concentration	6.82	<0.001
	Root vigor	Hormone	537.61	<0.001
		Concentration	2163.60	<0.001
		Hormone × concentration	52.25	<0.001
	MDA	Hormone	21.43	<0.001
		Concentration	672.86	<0.001
		Hormone × concentration	6.43	<0.001
	Ethylene	Hormone	10.50	0.003
		Concentration	691.43	<0.001
		Hormone × concentration	0.00	1.000
6 d	Primary root length	Hormone	738.11	<0.001
		Concentration	59,526.26	<0.001
		Hormone × concentration	825.47	<0.001
	Total root length	Hormone	93.51	<0.001
		Concentration	7658.11	<0.001
		Hormone × concentration	90.14	<0.001
	Lateral root number	Hormone	10.12	0.004
		Concentration	165.80	<0.001
		Hormone × concentration	0.68	0.646
	Root vigor	Hormone	40.74	<0.001
		Concentration	3883.03	<0.001
		Hormone × concentration	1.21	0.337
	MDA	Hormone	1.00	0.327
		Concentration	593.80	<0.001
		Hormone × concentration	0.40	0.844
	Ethylene	Hormone	6.25	0.020
		Concentration	541.05	<0.001
		Hormone × concentration	0.25	0.936

**Table 2:** Log–dose regression analysis of primary root length and ethylene under 6-BA and KT treatments.

Time	Hormone	Response	Slope	R^2^	*p*
3 d	6-BA	Primary root length	−0.882	0.989	<0.001
	6-BA	Ethylene	0.509	0.990	<0.001
	KT	Primary root length	−0.783	0.823	<0.001
	KT	Ethylene	0.509	0.990	<0.001
6 d	6-BA	Primary root length	−1.803	0.935	<0.001
	6-BA	Ethylene	0.520	0.986	<0.001
	KT	Primary root length	−1.630	0.972	<0.001
	KT	Ethylene	0.520	0.986	<0.001

## Data Availability

The original contributions presented in the study are included in the article; further inquiries can be directed to the corresponding author.
